# Confirmation of HCV viremia using HCV RNA and core antigen testing on dried blood spot in HIV infected peoples who inject drugs in Vietnam

**DOI:** 10.1186/s12879-018-3529-3

**Published:** 2018-12-04

**Authors:** Truong Tam Nguyen, Véronique Lemee, Karine Bollore, Hai Vinh Vu, Karine Lacombe, Xuan Lien Truong Thi, Que Anh Luong, Charline Dubos, Jean-Christophe Plantier, Huong Duong Thi, Didier Laureillard, Maud Lemoine, Edouard Tuaillon

**Affiliations:** 1 University of Medicine Pham Ngoc Thach, Ho Chi Minh City, Vietnam; 2 Pathogenesis and Control of Chronic Infections, INSERM, Université de Montpellier, CHU Montpellier, Montpellier, France; 3 Normandie Univ., CHU Rouen, Laboratoire de Virologie, Rouen, France; 4 Department of Infectious and Tropical Diseases, Viet Tiep Hospital, Hai Phong, Vietnam; 5 Department of Infectious and Tropical Diseases, Saint-Antoine Hospital, AP-HP, Sorbonne Universités, Université Paris 06, INSERM S 1136, iPLESP, Paris, France; 6 Pasteur Institute, Ho Chi Minh City, Vietnam; 7 Department of Public Health, University of Medicine and Pharmacy, Hai Phong, Vietnam; 8 Pathogenesis and Control of Chronic Infections, INSERM, Université de Montpellier, CHU Nîmes, Montpellier, France; 9 Department of Hepatology, St Mary’s Hospital, Imperial College, London, UK

**Keywords:** Hepatitis C virus (HCV), Human immunodeficiency virus (HIV), HCV core antigen, HCV RNA, Vietnam

## Abstract

**Background:**

Nucleic acid tests performed on blood samples collected on Dried Blood Spot (DBS) and detection of HCV core antigen (HCVcAg) are two approaches that may facilitate access to HCV diagnosis in low and middle incomes countries. In this study we evaluate HCV RNA and HCV antigen testing on DBS in HIV/HCV co-infected peoples who inject drugs in Vietnam.

**Method:**

One hundred and four HIV/HCV seropositive patients managed in outpatient care at the Haiphong Viet Tiep hospital were included in this study from February to March, 2014 (ANRS 12262 study).

**Results:**

Eighty-six subjects were tested positive for HCV RNA in serum, median (IQR): 6.9 log_10_ IU/ml (5.6–7.4 log_10_ IU/ml). Genotypes consisted of 57 G1 (69%), 3 G3 (4%), and 22 G6 (27%). HCV RNA was detected on DBS specimens in 79 out 86 subjects with chronic hepatitis C (sensitivity 92.5%; 95% CI: 85.1–96.9%). HCV RNA level on DBS and serum was moderately correlated (*r* = 0.24; *p* = 0.05) suggesting a degradation of HCV RNA due to transportation and storage conditions. HCVcAg was detected in 75/86 dB specimens (sensitivity: 87.2%; 95% CI: 78.3–93.4%), with a strong positive relationship between DBS HCVcAg and serum HCV RNA levels (*r* = 0.80; *P* < 0.0001).

**Conclusions:**

Quantification of HCVcAg on DBS appears to benefit from substantial stability under prolonged storage conditions but with a lower analytical sensitivity compared to DBS HCV RNA testing. Detection of HCV RNA on DBS is an interesting approach for confirming viral replication in HCV seropositive persons but the impact of pre-analytical conditions on the integrity of HCV RNA needs to be controlled.

## Background

Decisive breakthrough was recently achieved in hepatitis C virus (HCV) antiviral therapy but the limited access to reliable and low-cost HCV diagnosis tools is a key barrier in the global fight against HCV epidemic [1]. The World health organization (WHO) has recently incorporated HCV elimination in its 2030 agenda and has called for a 90% decrease in HCV incidence and 65% of HCV-related mortality [2]. In order to achieve these objectives, it is critical to simplify HCV testing in particular in high risk populations. However, HCV infection is frequently undiagnosed because the course of the disease is generally asymptomatic until end stage of liver disease. In low- and middle-income settings, where the burden of HCV is high, it is estimated that more than 95% of people are unaware of their HCV status [3, 4]. Screening for HCV infection is currently based on the detection of anti-HCV antibodies (HCVAb) followed by HCV RNA detection to confirm active infection. Rapid diagnosis test format provides a valuable alternative to laboratory tests for the scaling-up of HCV testing [4]. Over the last years whilst simple rapid diagnosis tests for HCVAb detection have been developed with two manufacturers fulfilling requirements for the WHO prequalification [5, 6], access to molecular assays is still limited in resource-limited countries. Poor laboratory capacities and logistical difficulties represent key barriers for the scaling testing and the treatment, particularly in rural areas and difficult to reach populations.

Serological methods assessing HCV core antigen (HCVcAg) can be surrogate assays to nucleic acid tests for detection of HCV viremia. HCVcAg detection can be less costly than PCR and may have a clinical utility in low and middle income countries [7]. Conditional recommendation has recently been published by WHO to consider the use of HCVcAg assays as a possible method to confirm HCV infection [4]. In this first global guidelines directed at low- and middle-incomes countries, the capillary sampling on blotting paper (DBS) is also considered as an easily transportable and simple alternative to peripheral venous sampling for confirmation of HCV infection based on RNA detection [1, 4]. In rural and resource-limited areas, DBS offer a simple transportable alternative to peripheral venous sampling and represents an appealing method for scaling up testing.

In Vietnam, the presence of anti-HCV antibodies has been reported between 1 to 6% of the general population and is dramatically higher (up to 90%) in people who inject drugs [8, 9]. The Vietnamese Ministry of Health has recently developed a national HCV guidance upon the release of the first WHO Guidelines on HCV care and treatment [10]. We previously reported a high proportion of advanced liver fibrosis among HIV-HCV co-infected peoples who inject drugs in Haiphong, Northern Vietnam [11]. In this study we evaluate the performance of HCV RNA and HCV antigen detection on DBS collected in Haiphong Viet Tiep Hospital and compared to serum PCR using open polyvalent PCR platform.

## Patients and methods

### Study population

The source population was HIV-infected outpatients reporting past injection or ongoing drug use who also participated in a cross-sectional study conducted at the Viet Tiep hospital in Haiphong between February and March 2014 (ANRS 12262). Eligibility criteria were: age over 18 years, positivity for anti-HCV antibodies and HCV RNA, HCV treatment-naïve status, ART initiated for more than 6 months, CD4+ T cells count over 200/mm^3^. Patients tested positive for HCV antibodies using both a rapid test (SD Bioline anti-HCV rapid test, Standard Diagnostics Inc., Korea) and laboratory test (Phamatech anti HCV EIA kit, USA) were prospectively enrolled after providing written consents. Sample size calculation was performed based on an expected sensitivity of 0.85 (95% acceptable lower CI: 0.70) using HCVcAg on DBS for identification of HCV chronic hepatitis, to control the design accuracy [12]. HCV RNA quantification and HCV genotyping were assessed as previously described [10]. Briefly, HCV RNA in serum was extracted using the MagNa Pure 96 system and quantified using a CE marked HCV kit (HCV Real-time Quant, Sacace Biotechnologies, Italy) on LightCycler 480 instrument (Roche, USA). HCV genotyping was performed for samples with HCV RNA level above 1000 copies/mL as previously described. HCV subtypes were identified using the Los Alamos Hepatitis C sequence (www.hcv.lanl.gov). Blood samples from HCV infected outpatients followed in the Montpellier University hospital and having provided informed consent were used for the validation of PCR and HCVcAg methods on DBS specimens (DC-2008-417). HCV RNA levels of HCV samples collected in France were quantified using the COBAS® AmpliPrep/COBAS® TaqMan® V2.0 assay (Roche), and genotypes were determined using the HCV Versant Genotype 2.0 assay (LiPA) (Siemens Healthcare Diagnostics, Tarrytown, NY). HCV genotypes consists of G1 (*n* = 18), G2 (*n* = 2), G3 (*n* = 9), G4 (*n* = 8), G5 (*n* = 1). The study protocol and written informed consent form were approved by the Ethic Committee of the Viet Tiep Hospital and the Institutional Review Board (IRB) of the Haiphong Medical Services (n° 01BVVT/HDKH). Then, the written informed consent was obtained from all participants before blood sampling.

### DBS specimen preparation

Fifty microliters of venous whole-blood collected in ethylene diamine tetraacetic acid tube was spotted onto the filter paper card (Whatman 903; GE Healthcare Europe, Freiburg, Germany). The filter paper was dried at ambient temperature, stored in an individual sealed plastic bag with a desiccant package at − 20 °C. DBS samples collected from the Montpellier University Hospitals were stored at − 20 °C until used. Samples from the Viet Tiep hospital in Haiphong were defrosted and transported at ambient temperature during 72 h, and stored again at − 20 °C for a mean duration of 18 months until HCV RNA and HCVcAg testing.

### HCV RNA testing on DBS specimens

HCV RNA were quantified in DBS using the HCV Generic assay (Generic HIV Viral Load, Biocentric, France) with a detection limit of 80 IU/mL using 0.2 mL of serum. For DBS elution, 2 punched disks with a 6 mm diameter was eluted into 200 μl of elution buffer (PBS, BSA 10%, Tween 0,05%) at 4 °C with gentle agitation for 1 h. The nucleic acid extraction was done using 140 μL of DBS eluate on the Qiacube apparatus, and the amplification - using the LigthCycler 480 thermocycler (Roche). An estimated input plasma volume of 2–3 μL per PCR were used instead of 40 μL per PCR on serum. Then, DBS samples collected in the Haiphong Viet Tiep hospital were tested for HCV RNA in the Montpellier University Hospital using the same procedure.

### HCVcAg testing on DBS specimens

HCVcAg testing was performed using an automated chemiluminescent microparticle immunoassay (Architect HCV antigen assay; Abbott Diagnostics, Chicago, Illinois) as previously described [13]. Briefly, a punched disk of 6 mm diameter was eluted into 1 mL of 0.05% Tween 20 PBS at 4 °C with gentle agitation for 60 min. A total of 20 μL of DBS supernatant was used for the assay.

### Statistical analysis

Continuous variables were described as median and inter-quartile range (IQR). HCV RNA concentrations were converted to Log_10_ values before performing statistical analysis. Relationships between quantitative variables were studied by means of Pearson correlation. Bland-Altman bias plots were used to assess the differences between the different assays and clinical samples. For each plot, mean bias and 95% confidence interval (95% CI) of the bias were calculated and the mean biases were compared using Student’s t-test. The agreement between the detection of DBS HCV RNA and DBS HCVcAg was analyzed using Cohen’s kappa test. All statistical analyses were done with MS Excel and Graphpad Prism 6.0 (GraphPad Software, Inc., San Diego, CA).

## Results

### Comparison of HCV RNA quantification on serum and DBS specimens

**Fig. 1:**
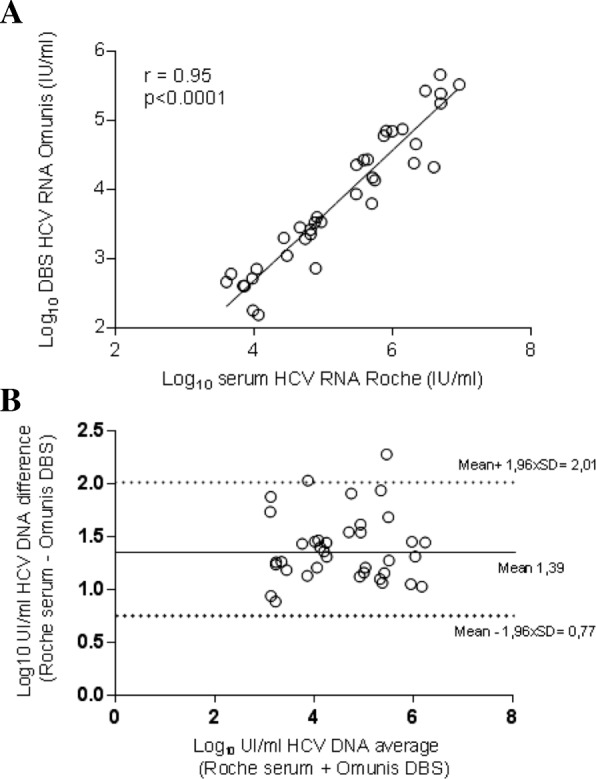
Regression analysis of HCV RNA levels and Bland-Altman plot of titer differences in paired DBS and serum samples collected in Montpellier University Hospital, France. **a** Linear regression analysis HCV RNA levels for 37 matched DBS–serum pairs. **b** Agreement between the HCV RNA quantification by plotting the differences between serum and DBS specimen averages of the two techniques using the Bland-Altman analysis

HCV RNA values on DBS specimens were compared to serum HCV RNA values quantified in matched serum samples collected in 37 HCV-infected patients followed in the Montpellier University Hospital, France. HCV RNA were detected in all DBS samples. The median IQR HCV RNA were 5.5 log IU/mL on serum and 3.8 log IU/mL on DBS elution buffer. We observed a good correlation between DBS using the Biocentric assay and serum HCV RNA using the Roche assay (Fig. 1a) (*r* = 0.9488; *P* < 0.0001). The mean difference (bias ± SD) between the results on DBS and the reference HCV RNA level was 1.391 ± 0.32 log IU/mL. Bland–Altman analysis shown that 36 (97%) results were seated within the 95% confidence interval (Fig. 1b). The limit of detection (LOD) for HCV RNA on DBS was estimated at 3000 IU/mL (data not shown).

### HCV RNA detection and quantification on DBS specimens collected from HIV/HCV co-infected patients from Viet Tiep hospital, Hai Phong, Vietnam

**Fig. 2:**
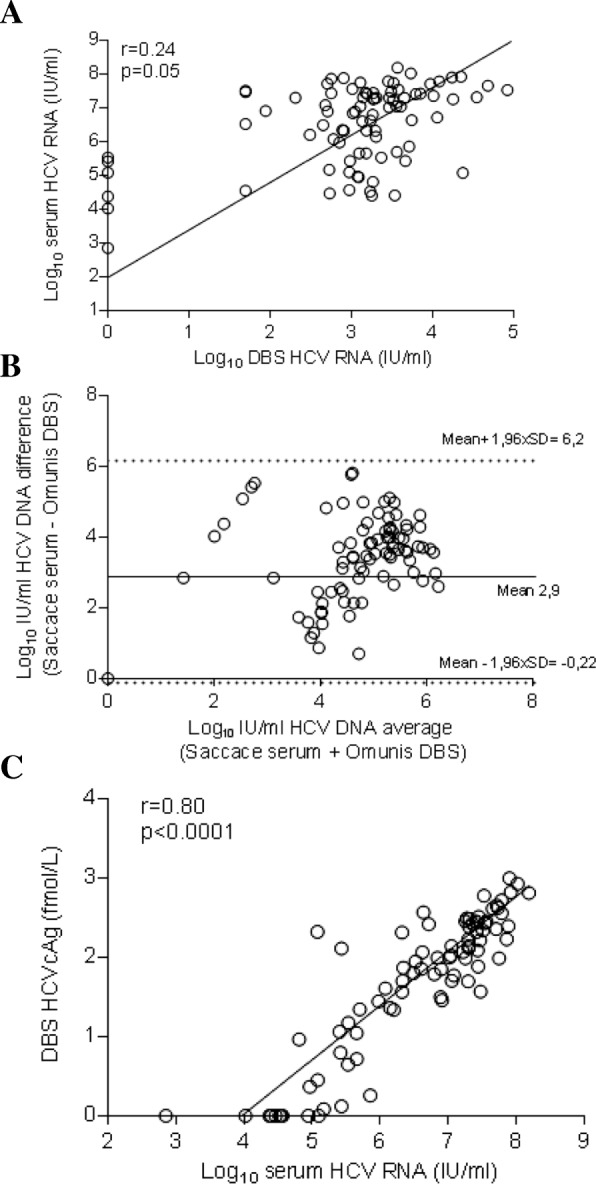
Assessment of HCV RNA and HCVcAg on DBS specimens collected from HIV/HCV co-infected patients care in Haiphong Viet Tiep Hospital, Vietnam. **a** Linear regression analysis HCV RNA levels for 86 matched DBS–serum pairs collected from subjects with chronic HCV infection. **b** Agreement between the HCV RNA quantification by plotting the differences between serum and DBS specimen averages of the two techniques using the Bland-Altman analysis. **c** Correlation between DBS HCVcAg and serum HCV RNA

One hundred and four patients were tested positive for HCV antibodies. DBS samples were available for 101 of them. Patients’ characteristics were summarized in Table 1. Eighty-six subjects (85%) were tested positive for HCV RNA in serum, median (IQR): 6.9 log10 IU/mL (5.6–7.4 log_10_ IU/mL). Genotypes were determined in 82/86 samples (95%) samples: G1 (*n* = 57), G3 (*n* = 3), G6 (*n* = 22). HCV RNA was detected in 79/86 dB samples (sensitivity 92.5%; 95% CI: 85.1–96.9%; negative predictive value 68.2%; 95% CI: 51.2–81.4%). Fifteen patients (15%) were tested positive for anti-HCV antibodies using rapid diagnosis test and laboratory method but were found negative for HCV RNA. None of above mentioned samples were found positive for HCV RNA on DBS (specificity: 100%; 95% CI: 78.2–100%; positive predictive value: 100%). Among seven specimens collected from viremic patients but tested negative for HCV RNA on DBS, the viral load in the matching serum ranged from 711 to 339,000 IU/mL (median 121,000 IU/mL; IQR: 10,600 to 261,000). Four DBS were positive for HCV RNA but with concentration seated below the lower limit of detection of the PCR assay (80 IU/mL), whereas in the matching serum the HCV RNA ranged from 35,500 to 32,600,000 IU/mL (median 16,360,000 IU/mL; interquartile range: 856,625 to 31,800,000). HCV RNA was successfully quantified in 75/86 samples (87%), (median HCV RNA: 1840 IU/mL of DBS elution buffer, IQR 957 to 4060). We observed a low correlation between DBS and serum HCV RNA measurements (Fig. 2a) (*r* = 0.24; *p* = 0.0135). Mean difference between HCV RNA values obtained with DBS vs. serum on Bland-Altman plot was 2.9 log IU/mL, with limits of agreement (mean difference ± 2 standard deviation) ± 3.28.log IU/mL (Fig. 2b). All the values were within the 95% confidence interval.

**Table 1:** Clinical characteristics of study participants

Characteristics	
Patients (total)	101
Sex Male/Female	97/4
Age years (IQR)	34.3 (31.2–40.3)
IV drug use (n)	72 (69.2%)
HCV and HIV diseases characteristics	
Fibrosis > F2 (n)	24 (23%)
CD4 T cell count (μl, IQR)	504 (357–624)
Nadir of CD4 T cells (μl, IQR)	82 (35–173)
2NRTI and NNRTI	97 (96%)
2NRTI and IP	5 (4%)
HIV RNA < 50 copies/ml (n)	97 (96%)

Median and interquartile range (IQR) were used for continuous variables

*SD* Standard deviation, *NRTI* Nucleosidique Reverse Transcriptase Inhibitors, *NNRTI* Non Nucleosidique Reverse Transcriptase Inhibitors

### HCVcAg detection and quantification on DBS specimens collected from HIV/HCV co-infected patients from Viet Tiep hospital, Hai Phong, Vietnam

DBS were tested for the presence of HCVcAg using the Architect HCV antigen assay. HCVcAg was detected on DBS in 75/86 (sensitivity: 87.2%; 95% CI: 78.3–93.4%; negative predictive value: 57.7%; 95% CI: 43.9–70.3%). All serum samples tested negative for HCV RNA were undetectable for HCVcAg in matched DBS samples (specificity 100%; 95% CI: 78.2–100%; positive predictive value: 100%). As shown in Fig. 2c, we observed a strong positive relationship between HCVcAg levels measured in DBS specimens and serum HCV RNA level (*r* = 0.80; *P* < 0.0001), regardless of the HCV genotype. Among the 11 dB samples collected from patient with chronic HCV infection but tested negative for HCVcAg on DBS, serum HCV-RNA viral load ranged from 711 to 88,600 IU/mL (median: 29300 IU/mL; IQR: 23,800 to 36,900 IU/mL). Of these samples tested negative for HCVcAg on DBS specimen but positive for serum HCV RNA, seven were positive for HCV RNA on DBS. By comparison, four DBS samples not tested negative for HCV RNA were found reactive for HCVcAg. A good agreement was observed between the HCV RNA and the HCVcAg Ag tests performed on DBS (Kappa coefficient: 0.65) in Table 2.

**Table 2:** Clinical performance and agreement of HCV RNA and HCVcAg detection on DBS

	Specificity % (n)	Sensitivity % (n; 95% CI)	Cohen coefficient
DBS HCV RNA	100% (15/15)	92.0% (79/86; 86.3–97.7)	
DBS HCVcAg	100% (15/15)	87.2% (75/86; 79.9–94.1)	
HCV RNA/HCVcAg agreement			0.65

## Discussion

Research evaluations are needed regarding novel laboratory techniques that would allow confirmation of HCV replication without the need for expensive laboratory equipment or trained personnel at the site of blood collection. Testing the feasibility, diagnostic accuracy, cost-effectiveness and clinical impact of HCVcAg or HCV RNA assays on DBS in field conditions remain some of the research gaps. In this study, the detection of HCV RNA using an open polyvalent PCR platform, and the detection of HCV core antigen on DBS confirmed the HCV replication in almost 9 out of 10 patients with chronic HCV infection. The nucleic acid tests on DBS provided a better sensitivity than the detection of HCVcAg, however, the HCVcAg antigen test on DBS appears to benefit from substantial stability under prolonged storage conditions.

HCVcAg detection may contribute to improve an access to HCV diagnosis in low- and middle-income countries. Blood collection on DBS, make possible to combine decentralized capillary blood collection with simple transportation from remote areas to regional laboratories [15]. Based on a systematic reviews and evidence summaries of the of the primary literature, recommendation was made by WHO to consider the use of DBS specimens as an option to facilitate an access to HCV antibodies and nucleic acid testing [16]. Good performance of nucleic acid tests on DBS have been reported for HCV RNA detection, with an overall sensitivity and specificity estimated to 96.0% (95% CI: 93.4–97.6%) and 97.7% (95% CI: 94.7–99.0%), respectively [17]. In this study we have concomitantly evaluated HCV RNA and HCVcAg on DBS in HIV/HCV co-infected people living in Vietnam. The sensitivity for HCV RNA detection on DBS collected in Vietnam were seated around the lower limit of the lower bounds values reported in previous studies [17]. Some samples with high serum HCV viremia were found undetectable for HCV RNA on DBS collected in Vietnam and we observed a low correlation for HCV RNA values between DBS and serum specimens. These DBS samples collected from the Hanoi Hospital were transported to France at ambient temperature, undertaken frozen-thawed cycles and were stored during a prolonged period. Thus, a partial degradation of HCV RNA can be suspected. DBS specimens are considered as generally stable over time, maintaining good accuracy in tropical conditions. However, degradation of RNA on DBS has been reported suggesting that HCV RNA levels can decrease over the prolonged transportation, long term storage at ambient temperature and freeze-thaw cycles [15, 19–21]. Our results confirm that assessment and control of the pre-analytical conditions remains critical for the accurate quantification of HCV RNA on DBS. In line with previous studies, we observed a high positive predictive value using HCVcAg detection on DBS [13, 18] showing that the assay is highly performing to confirm HCV replication. Laboratory performances of HCVcAg testing on DBS have been evaluated by Soulier et al. [13] but evaluation should be done in the field, in low-resource countries. High rate of HCVcAg detection on DBS was recently reported in HCV infected peoples who inject drugs in Tanzania, suggesting that HCVcAg on DBS may be an alternative to serum HCV PCR [18]. HCVcAg quantitation performed after 18 months of DBS collection was remarkably well correlated to serum HCV RNA level. This good correlation is in line with previous reports using serum specimens [20, 22], or DBS [13, 18] for HCVcAg quantitation. This observation suggests that HCVcAg is less susceptible to degradation than HCV RNA under the undesirable storage and transportation conditions that the DBS collected in our study underwent. HCVcAg detection on DBS can be a simple and useful alternative method to confirm HCV replication but the limit of serum HCVcAg detection estimated to about 3000 UI/mL HCV RNA [14, 22] is lower than serum HCV RNA assays. Furthermore, it is substantially reduced compared to the LOD of DBS HCV RNA,. Hence, the LOD of HCVcAg can be estimated equivalent around 10^5^ IU/mL HCV RNA [13, 18]. In our study all serum samples tested negative for HCVcAg on paired DBS have HCV RNA level below 10^5^ IU/mL. Even with such suboptimal sensitivity, the detection HCVcAg on DBS is able to confirm HCV replication in the majority of seropositive patients since the HCV RNA level is generally higher than 10^5^ IU/mL during the chronic HCV infection [23, 24]. Diagnostic algorithm based on HCVcAg detection has been proposed to directly identify chronic HCV infection [7, 18]. Predictive negative values over 90% [14] in blood but below 60% on DBS [13, 18] have been reported suggesting that the performances of HCVcAg testing may be insufficient as a one-step HCV screening and confirmation test when DBS are used. Furthermore, the HCVcAg test performed on the Abbott Architect platform is the only assay extensively studied and widely available [14]. The characteristics of this automated laboratory apparatus limit the access to this marker to well-equipped laboratories. No HCVcAg point of care tests are currently available to confirm HCV replication in subjects tested positive for HCV antibodies.

In conclusion, the quantification of HCVcAg on DBS appears to benefit from substantial stability under prolonged storage conditions but with a higher LOD than for HCV RNA on DBS. Detection of HCV RNA on DBS is an efficient approach for confirming viral replication in HCV seropositive individuals but should be accompanied by an assessment of the pre-analytical conditions observed during the transportation and HCV RNA preservation before the tests. Although the lack of qualification of the HCV tests on DBS specimens, and the absence of technological solutions adapted to resource-limited countries for HCVcAg testing are important limitations, one of the most feasible and cost-effective strategy to confirm HCV replication in many countries may be the collection of DBS for testing HCV RNA or HCVcAg in central locations.

## Conclusions

Quantification of HCVcAg on DBS appears to benefit from substantial stability under prolonged storage conditions but with a lower analytical sensitivity compared to DBS HCV RNA testing. Detection of HCV RNA on DBS is an interesting approach for confirming viral replication in HCV seropositive persons but the impact of pre-analytical conditions on the integrity of HCV RNA needs to be controlled.

## Abbreviations

ANRS: France REcherche Nord&Sud Sida-hiv Hépatites
DBS: Dried Blood Spot
HCV RNA: hepatitis C virus ribonucleic acid
HCVcAg: Hepatitis C virus core antigen
HIV: Human immunodeficiency virus
PCR: Polymerase chain reaction
RNA: Ribonucleic acid
WHO: World Health Organization
